# Getting it right when budgets are tight: Using optimal expansion pathways to prioritize responses to concentrated and mixed HIV epidemics

**DOI:** 10.1371/journal.pone.0185077

**Published:** 2017-10-03

**Authors:** Robyn M. Stuart, Cliff C. Kerr, Hassan Haghparast-Bidgoli, Janne Estill, Laura Grobicki, Zofia Baranczuk, Lorena Prieto, Vilma Montañez, Iyanoosh Reporter, Richard T. Gray, Jolene Skordis-Worrall, Olivia Keiser, Nejma Cheikh, Krittayawan Boonto, Sutayut Osornprasop, Fernando Lavadenz, Clemens J. Benedikt, Rowan Martin-Hughes, S. Azfar Hussain, Sherrie L. Kelly, David J. Kedziora, David P. Wilson

**Affiliations:** 1 Burnet Institute, Melbourne VIC, Australia; 2 Department of Mathematical Sciences, University of Copenhagen, Copenhagen, Denmark; 3 School of Physics, University of Sydney, Sydney, Australia; 4 Institute for Global Health, University College London, London, United Kingdom; 5 Institute of Social and Preventive Medicine, University of Bern, Bern, Switzerland; 6 Institute of Global Health, University of Geneva, Geneva, Switzerland; 7 Institute of Mathematical Statistics and Actuarial Science, University of Bern, Bern, Switzerland; 8 Institute of Mathematics, University of Zurich, Zurich, Switzerland; 9 Universidad del Pacífico, Lima, Peru; 10 The Kirby Institute, UNSW Sydney, Sydney NSW, Australia; 11 The World Bank Group, Washington DC, United States of America; 12 UNAIDS Myanmar, Yangon, Myanmar; Middlesex University, UNITED KINGDOM

## Abstract

**Background:**

Prioritizing investments across health interventions is complicated by the nonlinear relationship between intervention coverage and epidemiological outcomes. It can be difficult for countries to know which interventions to prioritize for greatest epidemiological impact, particularly when budgets are uncertain.

**Methods:**

We examined four case studies of HIV epidemics in diverse settings, each with different characteristics. These case studies were based on public data available for Belarus, Peru, Togo, and Myanmar. The Optima HIV model and software package was used to estimate the optimal distribution of resources across interventions associated with a range of budget envelopes. We constructed “investment staircases”, a useful tool for understanding investment priorities. These were used to estimate the best attainable cost-effectiveness of the response at each investment level.

**Findings:**

We find that when budgets are very limited, the optimal HIV response consists of a smaller number of ‘core’ interventions. As budgets increase, those core interventions should first be scaled up, and then new interventions introduced. We estimate that the cost-effectiveness of HIV programming decreases as investment levels increase, but that the overall cost-effectiveness remains below GDP per capita.

**Significance:**

It is important for HIV programming to respond effectively to the overall level of funding availability. The analytic tools presented here can help to guide program planners understand the most cost-effective HIV responses and plan for an uncertain future.

## Introduction

The question of how to prioritize investments across a range of public health interventions is central to health economics. Cost-effectiveness analyses were developed to address this question within an evidence-based framework, but their use has been limited, with investment prioritization in many countries determined by historical precedent rather than cost-effectiveness analyses[1].

Prioritizing investments across interventions that aim to combat an infectious disease is particularly difficult. Traditional cost-effectiveness analyses rely on an accurate estimation of both program costs and outcomes to calculate cost-effectiveness ratios. A single cost effectiveness ratio will be prepared for a given intervention in a given context, thus assuming constant or linear marginal outcomes for this intervention at all levels of expenditure. However, for the majority of infectious diseases, both costs and outcomes are likely to be highly nonlinear functions of program coverage[2–5]. This means that the cost-effectiveness ratio varies depending on the level of investment. Furthermore, interaction effects between different programs mean that the cost-effectiveness of any given program depends on the coverage levels of all other programs[6]. It is thus necessary to consider an entire response simultaneously, in order to accurately understand the cost-effectiveness of a single intervention. Notable examples of studies that have attempted this for HIV include a study of HIV programs in Vietnam[7], studies of harm reduction packages for people who inject drugs in different contexts[8, 9], and studies of Avahan programs in South India[10]. Examples can also be found for malaria[11, 12] and tuberculosis[13] responses. In all examples, synergies between interventions have been emphasized.

For this study, we investigated HIV programmatic priority setting in different HIV epidemics. We did this by conducting modeling research exercises with four case study epidemics. The case studies were based on public data available for Belarus, Peru, Togo, and Myanmar, and represent epidemics across four different continents, with different national income levels, HIV funding levels, and epidemic conditions. The Optima HIV model has been, or is being, used by each of these countries in separate studies to guide target setting or investment choices towards the country’s national priorities. This study uses the Optima HIV model but with public data, addressing standardized objectives to compare and contrast outcomes across different settings to draw principles for HIV investment decisions. Namely, we investigated the relationships between (a) the total level of investment in an HIV response, (b) the interventions that should ideally be included in an HIV response to minimize HIV-related disability-adjusted life years (DALYs), and (c) the cost-effectiveness of the HIV response in terms of cost per DALY averted.

We chose to focus on HIV investments for a number of reasons. Firstly, evidence suggests that there is some uncertainty over the levels of both international[14] and domestic funding[15] for HIV programming in coming years. This is especially true in lower- and lower-middle-income countries where HIV programming has historically been heavily donor-dependent. An investigation of the relationship between investment levels and cost-effectiveness is therefore timely[14]. Secondly, studies have suggested that the nonlinearities between investment and cost-effectiveness are likely to be particularly pronounced in the case of HIV[3], making it a good candidate for investigation. Thirdly, ongoing efforts support the maintenance of a unit-cost database for HIV interventions[16], and whilst this is expected to provide a wealth of data, it may also encourage users of these data to assume that linear cost functions apply to HIV interventions. Thus, it is again timely to investigate whether linearity is a reasonable assumption and if not, what other data should be collected to inform more sophisticated and accurate cost functions. Finally, the question of how many programs to maintain as part of an effective HIV response is relevant for all countries. The Joint United Nations Programme on HIV/AIDS (UNAIDS) advocates a combination prevention approach, with 25% of HIV budgets allocated towards primary prevention programs. This involves the simultaneous use of complementary behavioral, biomedical and structural prevention strategies[17], and there is evidence to suggest that these complementary strategies have been effective in reducing new infections. However, implementing a very wide range of combination prevention approaches may also run the risk of spreading investments too thinly over too many interventions. Some studies suggest this could be less effective than funding fewer interventions at greater levels[3].

The four case studies presented here aim to provide some insights in how to adapt the combination prevention approach to different epidemic types and funding landscapes.

## Methods

### Mathematical model

These analyses used Optima HIV (v2.5.1, available at hiv.optimamodel.com), a compartmental model of HIV transmission and disease progression linked to a programmatic response module. Optima HIV is capable of estimating the optimal allocation of a total HIV budget across different programs in order to produce the outcome best aligned with particular targets. For example, the model can be used to estimate the allocation of funding that would minimize HIV infections, or that would get as close as possible to national strategic targets[18].

### Overview of case studies

Optima HIV has been used by numerous national governments over the past six years to support analyses of their HIV responses[19]. Of these, we selected four case studies from four different regions of the world, with each country selected on the basis of the following criteria: (1) that the country had total HIV spending data (as reported in a National AIDS Spending Assessment or similar framework) from 2014 or more recent; and (2) that the country represented the largest possible share of the regional burden of people living with HIV. Applying these criteria, we selected Myanmar[20], Belarus[21], Togo[22], and Peru[23], which represent a range of epidemic types and funding landscapes. A summary of the main characteristics of each country’s HIV epidemic and response is provided in Table 1.

**Table 1:** Key characteristics of the HIV epidemic and response in Myanmar, Belarus, Togo and Peru. Acronyms used: PWID: people who inject drugs; FSW: female sex workers; MSM: men who have sex with men.

	MYANMAR	BELARUS	TOGO	PERU
**HIV epidemic**				
**People living with HIV (2015)[24]**	220,000	35,000	110,000	66,000
**Adult (15–49) HIV prevalence (2015) [24]**	0.8%	0.6%	2.4%	0.3%
HIV prevalence among key populations	PWID: 23.3% (2014)[25] FSW: 6.3% (2014)[25] MSM: 6.6% (2014)[25]	PWID: 14.2% (2013) FSW: 5.8% (2013) MSM: 6.2% (2013)	PWID: 5.5% (2014)[26] FSW: 16.8% (2014)[27] MSM: 16.6% (2014)[28]	FSW: 2.13% (2011)[29] MSM: 12.4% (2011)[29] Transgender women: 20.8% (2011)[29]
Characteristics	Greatest proportion of new infections among PWID and their partners	Greatest proportion of new infections among PWID and their partners	Greatest proportion of new infections among females 25–49. Among key populations, infections are highest among FSW and their clients	Greatest proportion of new infections among MSM and transgender women
**HIV response**	2015	2014	2014	2015
Total HIV investment	US$69m	US$20m	US$21m	US$92m
Total HIV investment per person with HIV	US$314	US$571	US$191	US$1,394
Domestically-funded share of HIV investment	12%	71%	25%	>99%
Share and percentage (%) of HIV investment allocated to targeted programs	US$51m (74%)	US$9.5m (48%)	US$12m (59%)	US$88m (96%)

### Model inputs and calibration

The epidemic model within Optima HIV was populated with country-specific behavioral, clinical, demographic and programmatic data from 2000 until the latest year for which data were available. Input data types and sources are summarized in Table 2. In Belarus and Togo the most recent data were from 2014, while in Peru and Myanmar the most recent data were from 2015. The epidemic model was calibrated to available data on HIV prevalence, population sizes, number of people on treatment, and number of PLHIV. Uncertainty estimates were generated around the model projections using an Approximate Bayesian Computation (ABC) algorithm[30], with prior distributions defined over two types of parameters: HIV prevalence in each population in the year 2000, and a population-specific scalar that applies to the time-varying and data-informed probability of transmitting HIV. By sampling from the prior distributions of these parameters, we obtained a set of possible epidemic outcomes at each point in time.

**Table 2:** Key parameters used to inform the transitions for the epidemiological model.

Transitions	Data types	Value
**Infection**	Sexual behavioral data (number of acts per year & probability of condom use with regular, casual and commercial partners)	Time-varying and population-specific sources and values for Myanmar[20], Belarus[21], Togo[22], and Peru[23]
	Injecting behavioral data (number of injections per year and probability of needle-syringe sharing)	Time-varying and population-specific sources and values for Myanmar[20], Belarus[21], Togo[22], and Peru[23]
	Intervention uptake (% of people accessing PrEP, circumcision, ART, OST and PMTCT)	Time-varying and population-specific sources and values for Myanmar[20], Belarus[21], Togo[22], and Peru[23]
	Per-act transmission probabilities	[31]
	Efficacy of interventions	[31]
	Partnership formation patterns	Sources and values for Myanmar[20], Belarus[21], Togo[22], and Peru[23]
**Diagnosis**	% of population tested for HIV in the last 12 months	Time-varying and population-specific, sources and values provided in reports for Myanmar[20], Belarus[21], Togo[22], and Peru[23]
**Treatment initiation**	Matched to number of people receiving ART	Time-varying and population-specific, sources and values provided in reports for Myanmar[20], Belarus[21], Togo[22], and Peru[23]
**CD4 progression**	Duration of acute infection	0.24 [0.10–0.30] years [31]
	Time to move from CD4≥500 to 350≤CD4<500	0.95 [0.62–1.16] years [31]
	Time to move from 350≤CD4<500 to 200≤CD4<350	3.00 [2.83–3.16] years [31]
	Time to move from 200≤CD4<350 to 50≤CD4<200	3.74 [3.48–4.00] years [31]
	Time to move from 50≤CD4<200 to CD4<50	1.50 [1.13–2.25] years [31]
**CD4 recovery on suppressive ART**	Time to move from 350<CD4<500 to CD4>500	2.20 [1.07–7.28] years [31]
	Time to move from 200<CD4<350 to 350<CD4<500	1.42 [0.90–3.42] years [31]
	Time to move from 50<CD4<200 to 200<CD4<350	2.14 [1.39–3.58] years [31]
	Time to move from CD4<50 to 50<CD4<200	0.66 [0.51–0.94] years [31]
	Time from treatment initiation to viral suppression	0.20 [0.10–0.30] years [31]
**CD4 progression & recovery on non-suppressive ART**	% moving from CD4>500 to 350<CD4<500 per year	2.60 [0.50–27.50]% [31]
	% moving from 350<CD4<500 to CD4>500 per year	15.00 [3.80–88.50]% [31]
	% moving from 350<CD4<500 to 200<CD4<350 per year	10.00 [2.20–87.00]% [31]
	% moving from 200<CD4<350 to 350<CD4<500 per year	5.30 [0.80–82.70]% [31]
	% moving from 200<CD4<350 to 50<CD4<200 per year	16.20 [5.00–86.90]% [31]
	% moving from 50<CD4<200 to 200<CD4<350 per year	11.70 [3.20–68.60]% [31]
	% moving from 50<CD4<200 to CD4<50 per year	9.00 [1.90–72.30]% [31]
	% moving from CD4<50 to 50<CD4<200 per year	11.10 [4.70–56.30]% [31]

### HIV responses and cost functions

The HIV response in each country was analyzed with the aim of understanding the impact of expenditure on programmatic outcomes. Programs were classified into either *targeted* or *non-targeted* programs. The former category consisted of all programs that directly affect one of the proximal determinants of HIV infection or disease progression (for example, condom distribution programs), while the latter was comprised of all crosscutting programs (including management, infrastructure, monitoring and evaluation, education and empowerment for young women, and other HIV care). For the targeted programs, cost functions relating program expenditure to the number of people covered and to behavioral or clinical outcomes were estimated. These were validated by national health departments of each country. Key parameter values needed to define each cost function consisted of the (a) average cost of reaching someone with the program at the current level of operations, (b) estimated maximal attainable coverage of the program, and (c) program impact in terms of behavioral or clinical outcomes (for example, the difference in HIV testing rates or condom usage for those covered by the program versus those not covered). Parameter values were allowed to vary uniformly over ranges within 10% of country-specified values, and by sampling from these distributions, we obtained a set of cost functions for each program.

These studies did not attempt to establish cost functions for non-targeted programs, since the impact of these programs on HIV incidence and AIDS-related deaths could not be directly quantified in the same way it can be for targeted programs.

### Cost-effectiveness calculations

For each country, we used Optima HIV’s optimization algorithm to estimate the minimum number of cumulative DALYs that would accrue between 2017 and 2030 under a set of annual budgets for targeted programs, ranging from 0% to 200% in 10% increments of the total amount available in the latest reported year (Table 1). The optimization algorithm consists of the following steps: firstly, an initial funding distribution is specified and the epidemiological outcome under this distribution is calculated. Secondly, the amount of funding allocated to one program is changed and the model is rerun under the new allocation of funding (normalized to preserve the total budget). If the outcome was improved, the new funding distribution is accepted; otherwise, the distribution from the previous step is retained. This second step is repeated (on subsequent iterations, the choice of program based on probability distributions learned from previous iterations) until the solution converges. Further details are provided in [32].

For each budget, we calculated interquartile ranges around the estimated cumulative number of DALYs by drawing 5000 samples from the joint prior distribution of the parameters of the epidemic model and the parameters of the cost functions. We also calculated the cost-effectiveness ratios (with interquartile ranges) for each possible budget, relative to a zero-investment scenario. We use a hybrid approach for calculating DALYs (as detailed in [33]), with disability weights given in [31].

## Results

**Fig 1:**
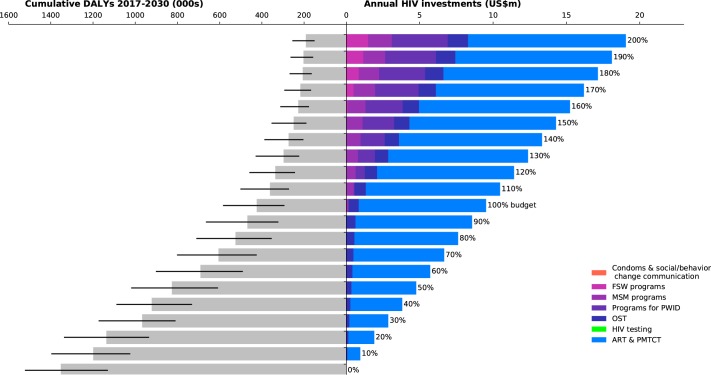
Investment staircase for Belarus. Illustrates the relationship between total (optimized) investments in the HIV response (right panel), and the overall outcome in terms of cumulative DALYs from 2017–2030 of that response (left panel; black bars indicate interquartile ranges).

**Fig 2:**
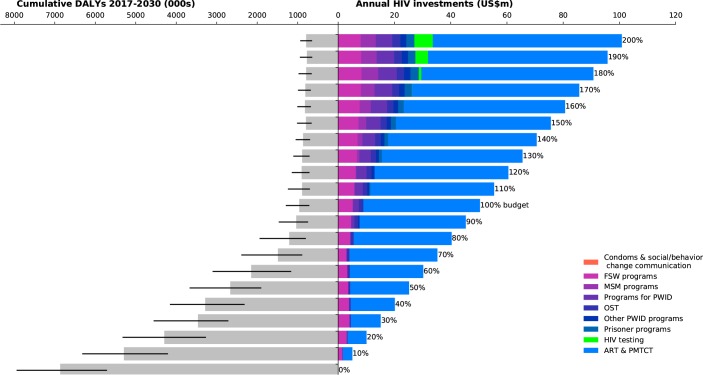
Investment staircase for Myanmar. Illustrates the relationship between total (optimized) investments in the HIV response (right panel), and the overall outcome in terms of cumulative DALYs from 2017–2030 of that response (left panel; black bars indicate interquartile ranges).

**Fig 3:**
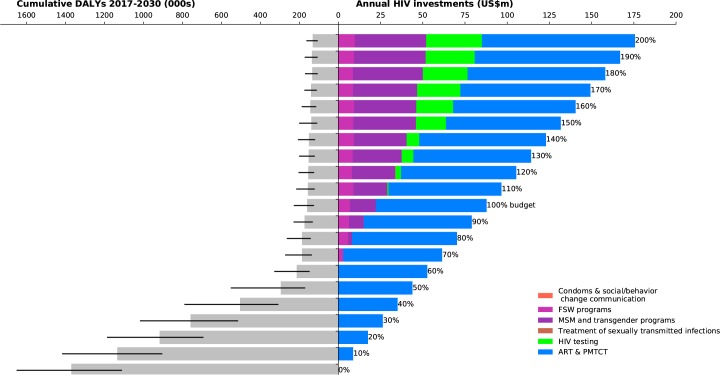
Investment staircase for Peru. Illustrates the relationship between total (optimized) investments in the HIV response (right panel), and the overall outcome in terms of cumulative DALYs from 2017–2030 of that response (left panel; black bars indicate interquartile ranges).

**Fig 4:**
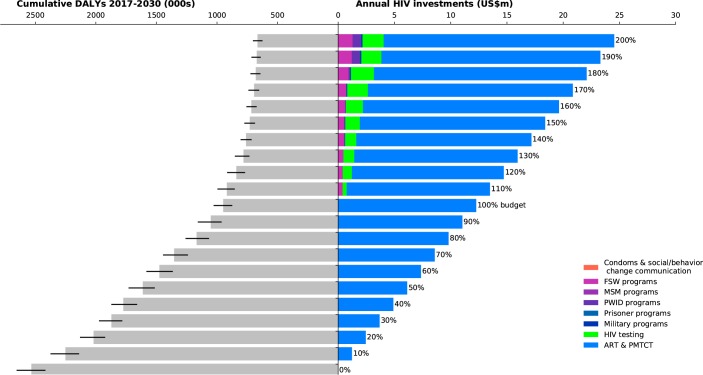
Investment staircase for Togo. Illustrates the relationship between total (optimized) investments in the HIV response (right panel), and the overall outcome in terms of cumulative DALYs from 2017–2030 of that response (left panel; black bars indicate interquartile ranges).

In Figs 1–4, we present results relating the level and allocation of investments in the HIV response to the outcome in terms of DALYs. We call this graphical presentation of results an *investment staircase*. Such investment staircases have been used in several Optima HIV studies [19], and are a useful tool for establishing investment priorities when budgets are uncertain.

Across all four case studies, we found that when very little money is available (for example, less than 50% of the amount available for targeted programs in the most recent year for which spending data were available), the optimal strategy is to focus on funding fewer programs in order to take advantage of economies of scale. Treatment programs, including both antiretroviral therapy (ART) and prevention of mother-to-child transmission (PMTCT) are the most important programs to retain, due to the important role that ART plays both in averting deaths and in lowering transmission risk. As more resources become available, a greater number of programs are included in the most cost-effective HIV response.

In Belarus (Fig 1), US$12m was spent on targeted HIV programs in 2014. According to the modeling analyses, if this same amount of funding were available annually between 2017 and 2030 and optimally allocated to minimize the number of cumulative DALYs, HIV treatment and opiate substitution therapy (OST) would be the highest priority programs to fund. With this allocation of the latest reported annual budget of US$12m, we estimate that the response would avert 65% [62%–74%] of DALYs over the period from 2017–2030 relative to no investment. Roughly the same allocation to the same priority programs would also be the optimal response at lower levels of funding. However, with less funding available, the predicted number of DALYs is higher. If more funds were available, the modeling analyses suggest that the optimal strategy would be to scale up these priority program areas and gradually introduce additional programs: investment in MSM programs would form part of the optimal HIV response if 120% of the 2014 budget were available, and investment in FSW programs if 170% of the 2014 budget were available.

In Myanmar (Fig 2), we found some similarities with Belarus. In both countries the HIV epidemic is primarily driven by PWID, and in both countries the modeling analyses found that if spending were optimized to minimize cumulative DALYs, then programs targeted to this population would be funded. However, there are also some notable differences. Investment in FSW programs was estimated to be highly effective in Myanmar and this program is therefore part of the optimal mix of investments even at very low funding levels. According to model estimates, if the 2015 budget for targeted programs were annually available each year between 2017 and 2030 and optimally allocated to minimize cumulative DALYs, it would avert 85% [83%–88%] of DALYs relative to no investment.

In Peru (Fig 3), we also found that investment in FSW programs form part of the optimal HIV response at low funding levels, but that further scale-ups in these programs will not achieve large gains. This may be attributed to the already-high rates of condom use in the FSW population. Investments in FSW programs in 2015 were around 7 times higher than in 2007, and despite low program coverage in 2007 (22%), condom use among FSW was already high at 72%[34]. On the other hand, further investment in programs targeting MSM and transgender women was estimated to be an effective use of resources. We found that investments in HIV testing programs for the general population should form part of the optimal HIV response only at higher levels of investment; at lower levels, a more targeted testing strategy (as is already included within programs targeted at key populations) is recommended.

In Togo (Fig 4), we found that if annual investments do not increase above their 2014 levels, the modeling results suggest that maintaining the ART and PMTCT programs would be the highest priority to minimize DALYs. Only around 32% of all PLHIV were receiving treatment in Togo in 2014[22] and providing treatment to those in need is a priority. If the annual budget increased by 10% over 2014 levels, investments in FSW programs and HIV testing would be prioritized by the model as part of the optimal mix.

In both Myanmar and Peru (Figs 2 and 3), there is a sharp decrease in the projected number of DALYs as annual investment in direct programs increase from 0% to 60% of the latest reported annual investment level. If investment in targeted programs were 50% of their levels in the latest reported year and optimally allocated to minimize DALYs, this would already avert an estimated 61% [54%–67%] of DALYs in Myanmar and 79% [67%–85%] in Peru relative to a zero-investment scenario. However further increases in investment are estimated to have a smaller marginal impact. By contrast, the decline in marginal impact for Belarus and Togo (Figs 1 and 4) is more gradual, but steady. In Togo, this is likely to be because investment in the HIV response is quite low, and so there is still opportunity for significant gains through increased funding. In Belarus, it is likely to be because the epidemic is increasing, and so higher levels of investment could assist in better controlling the epidemic.

The current state of HIV care and treatment in each country was an important factor in determining how investments in HIV testing programs should be prioritized. In all four case studies, there was a pool of people who had already been diagnosed with HIV, but had not yet been initiated on treatment. Our analyses found that HIV testing programs should not be scaled up until those already diagnosed had been initiated on ART. HIV testing programs were estimated to form part of the optimal mix when investment levels reached 110% of their most recent levels in Peru and Togo (Figs 3 and 4), 180% in Myanmar (Fig 2), and 250% in Belarus (not shown).

In Table 3, we present the cost per DALY averted for each country at levels of investment ranging from 0% to 500% of the level invested in targeted programs in the latest reported year. In each case we compare the set of all targeted interventions at different budgets to a scenario in which no investments in the HIV response were made and calculated the cost per DALY averted. For each country, the cost per DALY averted was estimated to increase with increased investment in targeted programs. However, even at higher investment levels, the cost per DALY averted remains below the per-capita GDP in each country.

**Table 3:** Cost per DALY averted by optimally allocated HIV responses at different budget levels in Myanmar, Belarus, Togo and Peru.

	MYANMAR	BELARUS	TOGO	PERU
**Per-capita GDP in 2015 (US$)[35]**	$1162	$5741	$636	$6027
**Cost per DALY averted (US$) [lower quartile–upper quartile]**				
*% of latest reported annual spending*, *optimally allocated*				
50%	$84 [$82–$93]	$127 [$126–$133]	$96 [$97–$98]	$571 [$524–$651]
100%	$120 [$106–$141]	$144 [$142–$159]	$112 [$110–$115]	$1064 [$865–$1266]
150%	$174 [$153–$209]	$181 [$171–$213]	$147 [$142–$153]	$1570 [$1270–$1874]
200%	$232 [$201–$279]	$230 [$211–$272]	$188 [$182–$197]	$2078 [$1671–$2482]
250%	$377 [$362–$397]	$297 [$266–$340]	$232 [$223–$243]	$2604 [$2090–$3107]
300%	$452 [$433–$475]	$349 [$310–$401]	$278 [$267–$292]	$3123 [$2499–$3730]
350%	$527 [$504–$553]	$403 [$358–$462]	$325 [$312–$342]	$3643 [$2930–$4357]
400%	$599 [$574–$630]	$456 [$403–$526]	$372 [$357–$391]	$4166 [$3338–$4973]
450%	$672 [$644–$707]	$512 [$450–$592]	$419 [$402–$440]	$4685 [$3748–$5594]
500%	$746 [$715–$785]	$566 [$498–$655]	$466 [$447–$490]	$5244 [$4191–$6221]
*Most recent annual HIV spending*, *allocated as in most recent year*				
100%	$125 [$117–$144]	$259 [$254–$279]	$147 [$146–$154]	$1124 [$1112–$1284]

## Discussion

We have presented results linking the total amount of investment in HIV responses to (a) the number of interventions estimated to constitute the optimal response (according to our mathematical model) and (b) the outcome (in terms of cumulative DALYs) associated with that response. For the four case studies that were considered here, some general principles apply. Specifically, we found that it is generally optimal to focus on a smaller number of HIV programs if budgets are highly constrained, and that ART should be prioritized. We also found some important differences in the way that cost-effectiveness changes as a function of total investment, which indicates that these kinds of analyses should be applied on a country-by-country basis in the context of the country’s health sector objectives and system capacity in order to draw the most relevant and useful messages.

We note some limitations to this study. Firstly, the results are only as reliable as the data upon which they were based. To the extent possible, we have accounted for the uncertainties in underlying data using appropriate statistical methods, and by reporting ranges around estimates. Secondly, the interventions selected by the model depend to a large extent on the objective that it being targeted. For this analysis, it was necessary to employ a standardized objective in order to enable meaningful cost-effectiveness calculations, and so we focused on finding the allocation of funding across different programs estimated to minimize the number of cumulative DALYs between 2017–2030. For practical policy purposes, however, each country will have different national priorities, and therefore the optimal allocation of funding across the HIV response may differ from that which we present here.

In making the connection between total investment levels and the number of programs to fund, we have employed investment staircases, which serve as a useful visualization tool for guiding prioritization of program maintenance or funding. In addition, the relationships between the level of investment in the HIV response and the overall (optimized) outcome of the response can serve as a basis to guide intra-regional or intra-disease allocations. This methodology has been employed in some studies already[36, 37], and will be detailed more explicitly in forthcoming work.

In order to carry out these kinds of analyses, it is essential to have detailed information to produce cost functions for specific HIV programs. The results of the four case studies presented in this paper were based on nonlinear cost functions that contained saturation effects once investment levels were very high. These saturation effects were intended to capture the fact that the costs of extending program coverage to the most difficult-to-reach groups are much higher. Capturing such nonlinearities required more detailed information on program costs than simple unit cost approaches allow.

The cost functions that were employed for these analyses were available thanks to the detailed allocative efficiency studies that had been conducted in partnership with national program experts. In general, in order to produce estimates of cost functions, we recommend that countries collect: (a) unit costs of programs; (b) total expenditure; (c) total coverage levels attained for each program; (d) the proportion of the total expenditure on each program allocated to targeted vs non-targeted (fixed) costs; (e) the estimated size of the target population, along with more detailed profiling that would enable estimates of the maximal feasible coverage levels of the target population; and, (f) supply- and demand-side constraints to expansion. Data such as these would permit cost-effectiveness analyses to be done more readily.

Cost-effectiveness analyses are essential to guide the large investments committed to HIV programming. Linking cost-effectiveness analyses to budget levels and program coverage is crucial to maximizing the impact of investments. This is particularly true in the current climate, when future funding may be uncertain.
